# Visions, expectations, and reservations in epigenetic editing: towards responsible innovation

**DOI:** 10.1186/s43682-026-00047-5

**Published:** 2026-03-13

**Authors:** Sophie van Baalen, Thomas Verra, Phil Macnaghten, Eline Bunnik, Michelle GJL Habets

**Affiliations:** 1 Rathenau Instituut, Den Haag, 2593 HW The Netherlands; 2 TNO, Den Haag, 2595 DA The Netherlands; 3 Knowledge, Technology and Innovation Group, Wageningen University, Wageningen, 6706KN The Netherlands; 4 Department of Public Health, Programme Medical Ethics, Philosophy and History of Medicine, Erasmus MC, University Medical Centre Rotterdam, Rotterdam, 3015 GD The Netherlands

**Keywords:** Epigenetic editing, Technology assessment, Responsible research and innovation, Epigenetics and society

## Abstract

In Science and Technology Studies (STS), scholars highlight the guiding role of expectations, promises, visions, and assumptions of scientists, shaping the development of research fields and technologies. Making these expectations and visions explicit, opens them to reflection and adjustment by diverse stakeholders, fostering more responsible and inclusive technological innovation. In this paper, we study the visions and expectations of scientists in academia and in industry (*n* = 19) in the field of epigenetics and epigenetic editing. Epigenetic editing is a technique that is used to modify the epigenome to gain regulatory control of gene expression. In the future, this could lead to the development of new biomedicines and biotechnologies to improve crops. We found that respondents broadly endorsed a vision of epigenetic editing as being milder and safer than genetic editing, and therefore more acceptable to the public, making it easier to embed epigenetic applications in society. However, respondents simultaneously questioned some of the underlying assumptions of this dominantly held vision and consequently expressed reservations. Although a limited number of studies have shown that epigenetic edits can be reversible and stable, uncertainty exists whether this is generalizable. Reservations are also linked to the possibility of heritability of edits, and to the level of complexity of (epi)genetic networks. In general, respondents were uncertain about the readiness of the technology to be currently implemented in the medical or agricultural sector. This contrasts with the current move of some companies to initiate clinical trials. To allow for more inclusive decision-making and co-shaping the research field and future applications, it is important to improve reflexivity among researchers and to enable public engagement. Having made these visions explicit, they become a topic for scientists, policymakers, and the public to reflect on and adapt or adjust, allowing for the co-creation of technological innovation.

## Background

Epigenetic editing is an upcoming field of practice that offers tools, such as CRISPR-dCas9 and zinc finger proteins, to provide regulatory control of gene expression [1]. Whereas epigenetic editing has been used by researchers to demonstrate the causal role of epigenetics in gene expression [1], more recently, it is emerging as a promising frontier in biomedicine [2–11]. There is a growing momentum for epigenetic editing, as illustrated by the explosion of peer-reviewed academic papers reporting successful epigenetic reprogramming of cells [12]. In addition, various spinoff biotechnology companies have emerged in recent years; there is progress in (the initiation) of clinical trials (8; https://otc.duke.edu/news/tune-therapeutics-moves-into-clinical-spotlight-with-tune-401-a-first-in-class-epigenetic-silencer-for-hepatitis-b/ (accessed 1 September 2025)) and recently, one of the leading companies in the field of epigenetic editing technology, Chroma Medicine, merged with Nvelop Therapeutics, which specializes in in vivo delivery technologies. (https://chromamedicine.com/2024/12/10/chroma-medicine-and-nvelop-therapeutics-unite-to-form-nchroma-bio-securing-75-million-to-accelerate-genetic-medicines/ (accessed 22 April 2025)). These companies promise “a new class of highly potent, durable, and specific genomic medicines [...] to revolutionize treatment of disease” (https://chromamedicine.com/ (accessed 24 January 2025)). Within the plant breeding sector, epigenetic editing has proven to be a useful tool to explore the relationship between the epigenome, gene expression, and plant adaptation [13]. In 

### Ethical and societal issues

The future, it may be deployed to accelerate plant breeding and enhance the ability of plants to cope with changing environments.

It has long been recognized that technology and innovations, besides bringing anticipated benefits to users (and society), often also have unintended, unforeseen impacts on other aspects of society, such as values, law, economy, or health. For this reason, various approaches have been established to evaluate technologies and innovations in view of their anticipated impact, such as Technology Assessment (TA) and its later versions that include Constructive TA (CTA), Participatory TA (pTA) and the ELSI (Ethical, Legal, Social implications) framework. Responsible Research and Innovation (RRI) builds on these frameworks with a guiding assumption that technology and its societal dimensions are mutually shaped and that ethical and societal concerns are an integral aspect of the ongoing scientific process. Prospective evaluation during the ongoing research process, based on anticipated consequences and on engagement with multiple stakeholders and the public, can result in the redesigning or reshaping of technologies in order to meet societal needs and to align with a wide range of values and ethical principles [14].

Scholars in the field of science and technology studies (STS) have attributed a specific generative role for *expectations*,* promises*,* and visions* in shaping development in science and technology [15], despite their speculative nature. Expectations and visions about the future of an innovation can guide activities of innovative actors; they can attract the interests of research communities and set the research agenda. And by providing legitimacy, they can attract public funding as well as foster private investments [15, 16]. For premarket applications in medical biotechnological developments, a bright future perspective can provide momentum [17]. Furthermore, when visions and expectations are shared amongst an increasing number of actors, the promises that are based on these visions and expectations can gradually turn into requirements and specifications of the new technology, shaping the further development of the research field and the technology itself [18]. Visions and expectations may become part of the mindsets of people outside the scientific community and inform public debates or policymaking [19].

### Co-creation

According to Mehnert and Grunwald, techno-futuristic visions are expressions of current states and processes which simultaneously impact or (re)shape the present [20]. For example, visions of human enhancement are embedded in an understanding of humans as bodies that can be upgraded by mechanical and artificial means [20, 21]. Visions and expectations of researchers in an emerging field can thus be analyzed to reveal present values and assumptions that are likely to shape future technological innovation – they are visions of what is believed to be a desirable future. By making explicit the visions that are co-shaping technologies, as well as their underlying values and assumptions, and the actors behind them, they become a topic for scientists, policymakers, and the public to reflect upon [20], and to adapt or adjust if necessary, thus allowing for the co-creation of responsible technological innovation. Including more voices may allow for incorporating broader foresight and impact assessments for new technologies, extending beyond their expected market benefits and risks [22]. This requires speculation about the course of the development of the technology and its possible future applications.

The field of epigenetic editing is in its infancy and applications have not yet been established or entrenched in clinical practice and/or agriculture. The field is currently in the midst of creating visions and expectations in order to attract international support and funding from both venture capitalists and public science funding bodies. In this study, we have conducted interviews to examine visions and expectations held by researchers working on epigenetic editing in both academic and industry settings, and to analyze their (often tacit) underlying assumptions and visions. We have also investigated how actors in the field describe the potential social and ethical implications of their research.

## Methods

This interview study was conducted within the Epi-Guide-Edit project, led by professor dr. Pernette Verschure. The Epi-Guide-Edit project is an interdisciplinary consortium that aims to provide robust rules to achieve long-term epigenetic reprogramming (https://www.rathenau.nl/nl/epi-guide-edit). This collaboration of various research universities, universities of applied sciences, the Rathenau Instituut and many companies, funded by the Dutch Research Council (NWO), focusses on agriculture and the medical field.

Respondents were purposely selected from the International Nucleome Consortium, a European interdisciplinary research consortium of nucleome scientists that aims to understand, predict and exploit the principles of genome organization in space and time to accelerate scientific breakthroughs (http://inc-cost.eu). Following initial recruitment, we used a snowball sampling approach to expand our sample, asking respondents to suggest other experts with relevant but different expertise. This method allowed us to obtain a wide range of visions and expectations for epigenetic editing. Recruitment was discontinued when data saturation was reached and thus when no new thematic content was found in transcripts of the respondents.

T.V., S.v.B. and M.H. conducted interviews between September 2023 and January 2024, using a topic guide based on extensive literature review and discussed within the multidisciplinary research team (Supplementary material 1). The open-ended questions revolved around (i) the developments, benefits, drawbacks, past and future breakthroughs, and challenges in the field of epigenetic editing (compared to other related techniques such as gene editing and epigenetic drugs); (ii) their expectations regarding the future of epigenetic editing; and (iii) ethical and societal aspects of epigenetic editing. The interviews lasted about 45–60 min and were audiotaped. All respondents gave written informed consent for their participation in this research. Most interviews were conducted in person and some online.

### Analysis

Interviews were transcribed *ad verbatim* using Happyscribe (https://www.happyscribe.com/), an artificial intelligence (AI)-based audio transcription platform. Transcriptions were verified by T.V. Using Atlas.ti, we analyzed the data thematically. Codes, concepts and themes were formed in the process of analysing the data. Themes were continuously evaluated within and across interviews and discussed by T.V., S.v.B. and M.H., resulting in a final thematic coding map.

## Results

Nineteen semi-structured interviews were conducted with scientists directly or indirectly involved in epigenetic editing research. Fifteen of these scientists were academic researchers, involved in translational or fundamental research, and four were employed by commercial companies. The respondents were involved in biomedical research (*N* = 14), plant science (*N* = 4), and in microbiology (*N* = 1) (see Table 1).

**Table 1:** Characteristics of respondents of our qualitative study

	Respondents
Female	6
Male	13
University	15
Businesses	4
Medical field	12
Plant breeding field	5
Micro organisms	1
The Netherlands	6
Ten European countries	12
US	1

Below, we set out our findings. We start by describing the dominant vision of researchers on epigenetic editing and its underlying assumptions, held by the majority of respondents. This dominant vision is simultaneously questioned to different degrees by the majority of respondents, who claim that the assumptions underlying the vision cannot be backed up yet with scientific evidence. Moving on, we describe how the views of the technology itself influence expectations of future applications. Last, we discuss the main ethical and societal issues raised by respondents.

Before addressing the dominant vision, we would like to draw attention to the finding that many respondents commented on how epigenetic editing is an important technical innovation and a great research tool that enables them to examine the complexity of gene regulation and gene expression. This in turn offers potentials for new knowledge on normal development as well as disease etiology, and on fundamental epigenetic mechanisms. It allows for the finding of causal connections in regulatory gene networks beyond mere correlations, and for unraveling the sequence of cascade events. Another benefit of epigenetic editing in research context which was mentioned in the interviews is that it enables the modulation of gene expression without requiring precise knowledge of the specific nucleotide changes needed to enhance promoter activity. By introducing targeted modifications to chromatin-associated proteins, this approach increases the accessibility of regulatory regions, thereby facilitating transcriptional upregulation. Most also foresaw a future for epigenetic editing as a medical treatment, although they differed in how they foresaw this future and its timeframe. Regarding plant breeding, respondents were more hesitant for reasons described in Section ‘Visions for the agricultural field’.

### Dominant vision of a future with epigenetic editing

The development of epigenetic editing was seen by our respondents as a safe alternative or additional tool to modify gene expression, which solves some of the problems and challenges currently associated with gene editing. For example, one of the challenges of gene editing technologies is the fact that inducing DNA-breaks, necessary to alter the DNA sequence, can lead to an unstable host genome. Because epigenetic editing does not cut the DNA strands but instead binds to a DNA sequence to block or stimulate the transcription of genes, it was assumed to be a safer alternative.

Another major challenge for gene editing, according to respondents, is off-target DNA breaks, where CRISPR-Cas9 (or any of the other mechanisms used to alter the DNA sequence) cuts unintended sections of the host genome. This can lead to non-specific, undesirable, and possibly even harmful changes to the genome. Although epigenetic editing technologies, such as CRISPR-dCas, also create off-target effects, they were viewed as less problematic – and thus safer to patients - because they do not coincide with DNA-breaks. A third argument why epigenetic editing is considered to be safer than gene editing lies in its expected reversibility: epigenetic changes in gene expression can in principle be reversed to their original states, which will be important when used in human/living beings, should serious side effects occur. The assumption is that the accompanying side effects will disappear along with the reversed edit. Epigenetic editing was also envisioned as *milder* or *more subtle* than gene editing. One reason for this lies in the proposition that changing the epigenome is a natural process that continuously takes place in the cell. It is therefore considered a lesser disruption of the processes in the cell than gene editing. A further argument mentioned by respondents is that the *effects* of epigenetic editing are milder than those of gene editing because rather than a binary on and off switch, or simply introducing or eliminating genetic functions, epigenetic editing is envisioned as a ‘thermostat’ with which gene expression can be fine-tuned. Because of both these aspects – it being considered a natural process in the cell, and more subtle – epigenetic editing is seen as more controllable and milder. However, respondents acknowledge several challenges common to gene editing and epigenetic editing, besides the already mentioned off-target binding by the guide strand; for example, there are difficulties delivering the large dCas9 protein into cells, and difficulties targeting the construct to specific organs.

Respondents also mentioned the benefits of epigenetic editing compared to epigenetic drugs: a class of therapeutic drugs that target epigenetic enzymes that regulate modifications to influence gene expression. These drugs were viewed to lack specificity, and to lead to many off-target effects [10]. We did not inquire after similarities and differences with prime editing and base editing, however one respondent mentioned the comparison spontaneously, and emphasized the benefits of epigenetic editing compared to prime-, base-, and gene editing.

*Certainly*,* you don’t have the translocation issue [with epigenetic editing]. Right. Nor the genetic instability because we’re not cutting DNA at all*,* unlike base editing or prime editing or gene editing. All of those are cutting at some level*,* whether it’s one or two strands*. (Interviewee 19)

### Questioning the dominant vision of epigenetic editing

Although respondents broadly endorsed the dominant vision of epigenetic editing as being milder and safer than genetic editing, they simultaneously questioned some of the underlying assumptions of this vision and consequently expressed reservations. Some provided their reservation unsolicited in the interview, whereas others expressed doubts only after being prompted by the interviewer. The reservations are linked to the assumed reversibility and stability of epigenetic editing, the possible somatic and intergenerational heritability, and the level of complexity of (epi)genetic networks.

### Reversibility and stability

The majority of respondents indicated that there remains a degree of uncertainty concerning the reversibility of epigenetic modifications. The degree of reversibility was seen as uncertain and as varying between the types of markers, the location in the genome, and the different technologies for epigenetic editing. Also, there were doubts expressed about the desirability of reversibility. Certainly, unintentional reversion by mechanisms in the cell is undesirable as it hampers the perceived ability of epigenetic editing to result in stable edits, and of being controlled, which is an important prerequisite for agricultural or medical applications. Some epigenetic changes are notably stable, practically permanent; however, respondents mentioned that scientists are (currently) unable to predict whether an edit at a specific site will be stable or not, and if so, for how long. Moreover, the stability or permanence of epigenetic edits may negate the promise of reversibility as a claimed advantage of epigenetic editing. Stability and reversibility are thus two sides of the same coin that make the non-permanent, or transient, character of epigenetic edits both beneficial and problematic for future applications. Research into the stability and reversibility of epigenetic edits is both needed and also challenging, as the following quote illustrates:

*We do not really know what makes epigenetic modifications stick around longer*,* or go away within days. […] It’s not easy to investigate because you cannot follow up with people for many years. This is something you need cohorts for.* (Interviewee 8)

### Heritability

Besides questions on the stability of an edit *within a cell*, respondents expressed doubts about the state of our current knowledge on its stability *during cell division* (mitotic heritability*)*, and on its stability *during reproduction* (inter- and transgenerational heritability).

For an edit to be heritable, it needs to be passed on through cell divisions to daughter cells, and it should thus not be removed during cell division. Respondents differed in their views on this mitotic heritability of epigenetic edits between daughter cells: some believed these edits will be passed on, others did not, and others still argued that there is currently not enough scientific evidence for arguing either way. For both medical and agricultural applications of epigenetic editing, mitotic heritability is a desirable goal, to maintain the desired effects of the epigenetic edits.

Besides the uncertainty about the mitotic heritability of edits during cell division, the respondents also expressed uncertainty about inter- and transgenerational heredity; about the extent to which it will occur and about the extent to which it should be thought of as desirable. Some respondents were concerned about the effects of epigenetic edits, when applied in humans, on their offspring. This uncertainty about somatic and transgenerational heritability is illustrated in the two quotes below:

*Epigenetics is something that occurs on top of my genomic sequence*,* but that can somehow functionally pass on*,* either from one generation to another*,* from a cell as it grows or from me to my offspring. […] From the mother cell to the daughter cells or from an organism to the offspring*. (Interviewee 3)

*Because the nice thing about that is*,* if the plant gets into the wild*,* it shouldn’t form issues when it gets out there. Because in theory*,* through generations*,* the epigenetic editing should be lost.* (Interviewee 15)

### Complexity

The final topic that led respondents to be hesitant about the claimed safety of epigenetic editing lies in the *complexity* of the genetic and epigenetic landscape, and the interaction between the two. Systemic complexity was seen to impede assessment of the predictability of the effects of epigenetic editing. Gene expression is controlled by many different factors and processes that are intertwined. In this system, cause and effect are not linear – changing an epigenetic state at one location in the DNA can cause a full cascade of unforeseen changes in the behavior of the cell. Especially when the targeted gene has many different functions, or when it is involved in the regulation of various other genes or genetic pathways, unexpected effects can occur.

Respondents argued that some of this complexity is caused by the dynamic nature of epigenetic processes and by their taking place in three dimensions: not only the methylation of the DNA sequence and the density of the chromosome impacts gene expression, but also the position of a gene in space. According to some respondents, this makes it (currently) impossible to unravel or predict the effects of epigenetic changes. Others mention how crosstalk between cells can modify the epigenetic landscape, another process that is not yet adequately understood by scientists. Respondents expressed the need for more detailed knowledge of genetic and epigenetic mechanisms to predict the effects of epigenetic editing.

Given the aforementioned uncertainties, the stated safety advantages of epigenetic editing technologies as compared to gene editing technologies were questioned. Due to a current lack of knowledge on the (epi)genetic system, the on- and off-target effects of epigenetic editing on living beings were seen as unpredictable and uncontrollable, and therefore potentially severe. Both can lead to changes in the expression of hundreds of other genes, potentially changing the behavior of the cell in radical ways.

To date, genetic and epigenetic networks remain largely uncharacterized and only partially elucidated:

*I think there are contexts where it can provide solutions for gene therapy: epigenome therapy in this case. You would need to have a very clearly solved epigenetic network that gives a very clear output. To be very honest*,* with the exception of imprinting*,* I cannot think of many that are such.* (Interviewee 3)

Even if some of these networks are characterized, the genetic and epigenetic make-up differs among individuals, making it hard to predict whether results in one individual can be used as a reliable indicator for results in others. While respondents partially agreed on the nature of the scientific uncertainties surrounding the reversibility, stability, and (somatic, inter- and transgenerational) heritability of epigenetic editing, as well as on the level of complexity of the genetic and epigenetic landscape, this shaped their view on the future of applications differently; the *consequences of those uncertainties for the further development of epigenetic editing applications* is interpreted in different ways, as we will demonstrate in the next Section.

### Visions for future applications

We identified three different visions among respondents on the future of epigenetic editing in medicine. The prevailing vision is ‘hopeful and modest’, whereas the two less common visions contrast each other, one being ‘careful’, and the other ‘a medical revolution’.

### Prevailing vision on future applications of epigenetic editing

Most respondents expected that epigenetic editing could be beneficial in the treatment of various diseases, the most mentioned being cancer. Some cancers are caused by epigenetic changes in the DNA of the cell which could be targeted by epigenetic editing as an innovative therapy. In addition, epigenetic editing could be used to enhance the efficacy of some of the current immunotherapies for cancers. For example, in CAR-T cell therapies, a patient’s immune cells are genetically edited ex vivo and placed back into the patients’ body to specifically attack cancerous cells. By implementing, in addition, the epigenetic editing of these CAR-T cells, these therapies could potentially be rendered more effective.

Respondents further expected that epigenetic editing could be an effective treatment for rare diseases caused by *epigenetic modifications*:

*You’ve got an epigenetic problem. You need an epigenetic solution*,* really. So*,* there’s a whole class of things*,* X-inactivation syndromes*,* that you could treat with those that seem ideally kind of suited to epigenetics.* (Interviewee 19)

Besides rare diseases caused by epigenetic modifications, some respondents also foresee a future for epigenetic editing-based therapies for rare diseases caused by *genetic modifications*, such as (mono)genetic diseases. Epigenetic editing can therefore exist side by side with gene therapy and other related technologies that together contribute to a range of medical applications that can enable a more personalized approach to the treatment of diseases.

In this prevailing vision, more knowledge is necessary for the field of epigenetic editing to move towards the clinic. When asked whether the current uncertainty on the dominant vision was problematic for moving forward, two respondents replied that this uncertainty also exists for other emerging therapies and drugs. They explained that it is common practice in medicine to not fully understand the mechanisms of novel therapies before the start of clinical trials, or even before marketing approval: taking risks can be necessary to develop treatments.

*If we think about classical medicine*,* we don’t even know the mechanism for a lot of drugs. We just know that they work. I think if we were demanding that epigenetic editing would be at such a degree of perfectionism that we wouldn’t do it unless we knew everything*,* we’re never going to do it. […] We don’t demand [full knowledge] for [the introduction of] other therapies. […] I think you have to be careful in terms of making sure that you’re not having any immediate adverse effects by testing*,* doing enough prior testing. This is why you have different stages of clinical testing. I think we know enough to test.* (Interviewee 9)

Upon confronting another respondent with their contradictory endorsement of the dominant view while also expressing doubts, they answered that epigenetic editing is reversible, and that long term side effects are therefore not to be expected.

Because of the high level of risk, and the current state of limited knowledge, many respondents argued that in early-stage clinical trials, it is important to enroll only patients with severe diseases that have no other treatment options. Almost all respondents envisioned that the first clinical trials in epigenetic editing would be a last resort option for patients.

### Other visions of future applications

In addition to this prevailing vision on applications for the medical field, we found two different visions expressed by a minority of respondents. As mentioned previously, some respondents were more cautious and doubtful that epigenetic editing would ever be safe for applications in a clinical context, due to the complexity of genetic and epigenetic systems. They foresaw a very limited role for epigenetic applications besides as an addition to gene therapy.

Basic researchers, who were not directly involved in translational research of epigenetic editing, in particular, expressed hesitancy about epigenetic editing in medical practice because of safety concerns. One interviewee argued that epigenetic editing could be less safe than conventional gene editing, because introducing epimutations could easily change the behavior of the cell.

*For instance*,* if you lose DNA methylation of repetitive sequences*,* that was a transposon*,* it starts being transcribed to be active to move from one part to the other part of the genome during the retro transposition process. We know that in some cases it can induce translocations*,* or it can produce cancer hijacking. It can be copied from one enhancer; it can be passed next to an oncogene for instance.* (Interviewee 1)

The interviewee also points out that as soon as epigenetically edited cells are in the body, the cells cannot be controlled by the scientists anymore, which might be dangerous for the patient. Scientists have developed genetic circuits designed to trigger cell death under specific conditions, so-called kill switches, making the intervention safer. However, selection pressure will select for cells with (random) mutations that overcome this cell death function. Reliable kill switches are therefore needed.

At the other end of the spectrum, a small minority of the respondents had high expectations of epigenetic editing for medical purposes. They predicted that epigenetic editing, together with gene editing and other techniques, will open up a future where medicine becomes more personalized and precise. These respondents envision a new range of epigenetic editing applications in medical practice that are fundamentally different to gene editing applications. Unlike gene editing, epigenetic editing does not cut the DNA, and in theory, this makes the modification of gene expression at multiple DNA locations feasible. This enables respondents to imagine other applications such as the treatment of autoimmune diseases or the treatment or cure of chronic illnesses such as diabetes, high cholesterol or Alzheimer’s disease.

*There is the ability to target multiple genes in order to get kind of a more complex shift*,* even turn the whole identity of a cell from*,* say*,* like a lymphoid*,* kind of a blood precursor*,* into something really specific*,* like a killer cell or something*,* or an effective cell in the immune system. Whereas the people have messed with that*,* with stem cells and transcription factors in a kind of scattershot way*,* we’ve really now kind of got a Rosetta stone for understanding how the body does that already.* (Interviewee 19)

In line with those potential future applications, some respondents imagined epigenetic editing as a possible remedy for aging. They imagined the possibility to reverse the effects of growing older, for example by using epigenetic editing-based creams to rejuvenate the skin or shampoo to reverse hair loss. Other possible medical applications that were mentioned by respondents included cures for serious viral infections such as HIV and Hepatitis-B and the development of antibacterial drugs based on epigenetic editing. One of the respondents with these high expectations of a future with epigenetic editing, when confronted with the fact that scientific uncertainty on the dominant vision remained high, replied that a future with gene editing was much more scary.

### Visions for the agricultural field

Respondents from the plant breeding field indicated that epigenetic editing was not (yet) a technology being developed for future commercial purposes in plant breeding. Moreover, they did not really foresee a future where epigenetic editing would play a major role. The most important reason for this was the lack of stability which makes epigenetic editing a less attractive option compared to genetic modification (including gene editing) in plants. For companies in the business of developing new plant varieties, it is very important that epigenetic modifications are stable over multiple generations and over different environmental conditions.

*If you have changed DNA methylation*,* and under your greenhouse conditions [they are] all stable*,* then it all works fine. But if you then bring this to the field with all its sensitivity to climate and you name it*,* yes then it remains to be seen. Yes*,* and that is also a huge challenge.* (Interviewee 10)

One advantage of epigenetic editing for human applications compared to gene editing, according to interviewees, is the fact that there may be fewer off-target effects since the cell can reverse these. However, off-target effects are less concerning in plant breeding due to the possibility of eliminating unwanted changes through repeated backcrossing with parent plants. Epigenetic editing was therefore not seen as a profitable avenue for industry, and respondents did not foresee industry (heavily) investing in the development of epigenetic editing technologies and possible applications.

Some respondents did mention a few hypothetical agricultural applications of epigenetic editing that would have a large impact on the field, if they could be developed. One example is the use of epigenome editing to develop plant species to become more adaptive to changing environments. Scientists might be able to develop plant varieties that can ‘activate’ modified gene expression under certain conditions; for example, a plant variety that only expresses genes involved in resistance to a certain disease when the disease is present. Another example where epigenetic editing could bestow major benefits over gene editing for plant breeders, is in the editing of traits involved in flowering. Flowering is partly epigenetically controlled and is the basis of all seed production. Being able to epigenetically induce the flowering process would give plant breeders a strong tool, because they would be able to reduce the breeding process by allowing plants to flower earlier and/or more often than is naturally the case.

### Ethical and societal issues

During the interviews, we asked respondents about their views on ethical and societal issues regarding future applications of epigenetic editing, as we believe that moral experience stemming from practice can enrich the ethical discussion as conducted in the literature. However, our findings did not reveal significant ethical concerns directly arising from such moral experience. Instead, the responses centered on more practical considerations.

Most respondents mentioned the expected high costs of medical applications of epigenetic editing – in line with the high prices for gene therapy. High prices are likely to put pressure on healthcare budgets, that could be spent more effectively otherwise. Moreover, high prices may lead to injustice: not all citizens, or all nations, might be able to afford these personalized treatments, leading to increased inequality.

*The risk here is that if we just focus all our attention and effort*,* money in developing this kind of super personalized approach*,* we’re not going to put effort into the development of other therapeutic options. In the end*,* it is not fair for the people who cannot pay for this*,* right?* (Interviewee 1)

Also, in the field of agriculture, expensive epigenetically edited crops might lead to (increased inequalities) between farmers in high- and low-income countries and to an increased dependence of small farmers on large, multinational companies. Although respondents foresaw that the price of epigenetic editing technologies will be high at the onset of marketing, they also expect that over time, prices will drop, because of their broad range of possible applications. They emphasized that other (biomedical) technologies are also initially expensive but tend to drop in price later.

Another ethical concern mentioned by respondents is the use of germline epigenetic editing. Respondents mentioned the issue of germline epigenetic editing as ‘ethically controversial’ in humans, but they reckoned that it would be very difficult or impossible to achieve, because epigenetic information is naturally reprogrammed twice during the early development of an embryo.

In the agricultural field, respondents also mentioned the risk that epigenetically modified traits might enter the wild population, albeit that this risk might be less for epigenetic editing versus gene editing, because epigenetic edits will ‘fade out’ over time.

*If you think about seeds and agriculture and kind of cross pollination and kind of competition with the natural wildlife*,* I think with epigenetic editing the very same can happen. It’s on us to show that it doesn’t. (Interviewee 17)*

A few respondents objected to non-medical uses of epigenetic editing, such as epigenetic doping, cosmetic use, reversing the effects of aging on appearance, or selecting favorable characteristics of offspring. Moreover, respondents were cautious towards the topic of enhancement, of pushing the boundaries of what is means to be human, and towards do-it-yourself uses of epigenetic editing.

*Now we’re doing genome editing for disease. We can do this. If people kind of realise*,* hey*,* this works. There is the risk of people*,* or*,* I know*,* there will always be the push for people to say*,* oh*,* actually*,* hey*,* I want a baby with blue eyes to become more kind of aesthetic and frivolous. And I think epigenetic is no different in that sense.* (Interviewee 18)

Another issue expressed by participants concerns the speed at which the field moves forward. If it moves too fast, there may be repercussions for the field, similar to those for the field of gene therapies in the 1990s:

*I feel that the same problem might come up with epigenetic editing when people are rushing it too quickly. It may be*,* it may get a bad reputation too soon and then be killed before it can be used properly.* (Interviewee 8)

However, if it moves too slow, it might lose momentum; it is important to sustain financial backing to deliver on the promise fast. Moving too slow may also lead to disappointed patients and citizens, especially if the technology is ‘hyped’ to the broader public, giving them too high expectations of the technology.

Finally, many respondents emphasized the societal issue of public acceptance of epigenetic editing. Because of the dominant vision among scientists of epigenetic editing as a safer and milder alternative to gene editing, some expect that the wider public will more readily accept clinical and agricultural applications of epigenetic editing. The envisioned benefits of epigenetic editing over gene editing are entangled with assumptions about public acceptability:

*Yeah*,* we’re not changing the genomic sequence*,* so public perception is a little bit easier with that because you haven’t edited the DNA. It’s not genetically modified. It’s the exact same genome. You just change the way that it’s being folded. […] We could have a system that’s a bit more publicly friendly or more publicly accepted. Highlighting the reversibility and the fact that it’s not changing things forever.* (Interviewee 11)

In contrast, other respondents worried that because the public will associate epigenetic editing with genetically modified organisms, they will dismiss epigenetic editing, especially because it will be difficult for the public to distinguish between gene editing and epigenetic editing. Whether respondents foresaw a positive or negative response from the public, they all positioned public perceptions of epigenetic editing as following in the tradition of public perceptions of genetic modification. In general, scientists believe that citizens are dismissive of genetic modification due to (scientifically unsupported) risks. Surprisingly, it is mostly medical scientists who believe that the benefits of epigenetic editing will change the public’s mind on genetically modified crops. Scientists in the agricultural field were more cautious. A solution put forward is to educate the public in the biological mechanisms of epigenetics.

Some respondents also expected less resistance towards clinical trials among patients with severe, untreatable conditions, such as cancers or genetic diseases that seriously affect young children. Subsequently, positive results from early clinical trials were believed to be able to pave the way for other applications, such as treatments for other diseases and wider commercial use.

## Discussion

This study explores researchers’ expectations, visions and assumptions on epigenetic editing. It shows that scientists see this emerging technology as a great research tool that enables them to examine the complexity of gene regulation and gene expression. The interviews also reveal a dominant vision of epigenetic editing as a safer and more acceptable alternative technology to gene editing and as a safer and more accurate alternative to epigenetic drugs. This view is based on the potential of epigenetic editing to solve some of the current challenges with gene editing [23]: epigenetic editing does not require DNA-breaks (avoiding genomic instability), its effects are believed to be reversible and not heritable between generations, and any off-target impacts, if they occur, are less concerning as they fade over time. Additionally, because in nature, epigenetic states of the DNA are constantly switching, epigenetic editing is seen as mimicking natural cellular processes that regulate gene expression in a subtle manner, and therefore is seen as less unnatural or ‘milder’ than gene editing. As a result, many respondents believe epigenetic editing will be more acceptable to the public. Although, we have mainly interviewed scientists and business experts from (ten different) European countries, and one from the United States, limiting our results to this region, we believe it is more broadly held, because both the vision and its underlying assumptions resonate with how epigenetic editing is presented and framed in the scientific literature [24–28]. It is also reflected in recent advances in translational research and development. However, the expected acceptability of the public towards epigenetic editing may be region-specific, as Europeans still express reservations towards genetically modified organisms. This may indeed be the reason why public acceptability is part of the dominant vision.

The controversy and public unease on genetically modified crops (GMOs) in Europe in the nineties, has resulted in the exclusion of GMOs in most European agriculture - while worldwide there has been steady growth in the area covered by GM crops. Although social scientists have observed that this controversy was due to factors such as the restricted scope for public and stakeholder involvement in the regulation of GM crop technologies, and the lack of formal consideration of non-scientific socio-economic or ethical considerations in assessment processes, natural scientists still believe that the controversy arose as a result of a misplaced misunderstanding, fear, and misinformation rather than an articulation of legitimate societal values [29]. Emphasizing anticipated public openness to epigenetic editing may be understood as part of the broader effort to promote the technology to policymakers and industry stakeholders, especially for those respondents that believe the public opinion will be constitutive for a future for epigenetic editing.

### Dominant vision

Interestingly, and perhaps not surprising, respondents questioned the dominant vision of epigenetic editing. The vision is, after all, based on an idealized version of epigenetic editing, and one that overlooks the inherent messiness of scientific practice including the ambiguity of experimental results, the challenges of interpretation, and the iterative, often chaotic nature of discovery. Such rhetoric reflects more an aspirational vision of science rather than its lived reality, where uncertainty and complexity are pervasive, especially in emerging fields, and especially in biology, that is inherently messy and complex. Scientists obviously are aware of this reality of science, leading some to mention the uncertainties that prevail on reversibility, stability and heritability, for some unsolicited, for others upon prompt. For these reasons, many respondents consider it desirable that more scientific knowledge is needed on the epigenome before moving to the clinic, even though the first clinical trials are ongoing.

The fact that the common thread in the field – that epigenetic editing is safer than gene editing – is simultaneously questioned by the field, and moreover, that both views can be held by individual scientists concurrently, is an important finding, as it questions the false sense of security that the dominant vision may provide [30], and highlights the need for serious reflection if epigenetic editing is to be developed in a responsible manner.

In September 2024, OMEGA Therapeutics announced the successful completion of their first clinical trial studying the downregulation of the expression of the oncogene c-MYC using epigenetic editing in 24 trial participants. Despite the company’s success in providing a proof-of-mechanism validating epigenomic controllers as a new class of medicines, they filed for bankruptcy in February 2025 (https://www.fiercebiotech.com/biotech/flagships-omega-sinks-toward-bankruptcy-lays-staff-13-months-after-inking-novo-obesity-pact (accessed on 17 October 2025)). Tune Therapeutics announced the start of their clinical trial on ‘an investigational epigenetic silencing therapy designed to treat chronic Hepatitis B (HBV)’ (https://tunetx.com/tune-therapeutics-moves-into-clinical-spotlight-with-tune-401-a-first-in-class-epigenetic-silencer-for-hepatitis-b/ (accessed on 1 September 2025)). The trial is currently recruiting human research participants (https://clinicaltrials.gov/study/NCT06671093 (accessed 1 September 2025)). Besides these two companies, several other venture-capital backed biotechnology companies are developing epigenetic editing as a platform technology [31]. Platform technologies are well-defined, standardized, replicable processes for consistent, high-quality production of therapies, for which the US Food and Drug Administration (FDA) has issued draft guidance in May 2024, to facilitate and expedite the evaluation and marketing authorization of drugs or biologics that utilize that platform technology (https://www.fda.gov/regulatory-information/search-fda-guidance-documents/platform-technology-designation-program-drug-development (accessed 1 September 2025); [32]). Despite the first clinical trials being underway, the majority of our respondents were skeptical about the readiness of the technology and felt uncertain regarding the envisioned key characteristics of epigenetic editing.

Due to the limited knowledge and the many uncertainties, respondents in our study expect that the first clinical trials of epigenetic editing-based therapies will focus on last resort therapies for cancer patients, which expresses their belief that the risks are still high. In contrast, a preclinical study that has garnered widespread media coverage, and is seen as an important step towards clinical translation, is the reduction of plasma cholesterol levels witnessed in mice and non-human primates with the goal of replacing statins for the treatment of high cholesterol [33, 34]. In an interview, following the publication of promising results in mice, the founder of Chroma Medicine, framed this ‘one-and-done’ epigenetic editing targeting cholesterol as a promising substitute for pill-taking for human patients [35]. Before filing for bankruptcy, OMEGA Therapeutics had announced to refocus its efforts and create an epigenomic editor to treat obesity in collaboration with Novo Nordisk (https://www.flagshippioneering.com/news/press-release/omega-therapeutics-announces-research-collaboration-with-novo-nordisk-to-develop-a-novel-therapeutic-for-obesity-management (accessed September 18, 2025)). A mismatch thus exists between the current direction of medical developments in the private sector – which seems focused not so much on severe diseases or unmet medical needs, but on common, actionable medical problems such as high cholesterol and obesity, – and academic scientists who seem more hesitant about the readiness of the technology. This mismatch may be due to the incentive of companies to move fast in other to be the first to claim success [36]. Spin offs and startups are often financed with venture capital and must therefore demonstrate their commercial viability withing a few years. Venture capital (VC) funds invest pooled capital in high-potential startups in exchange for equity, seeking high-risk, high-reward opportunities that can yield strong returns through a future sale or when the company goes public.

But also in academia, there is a high level of competitiveness and pressure to deliver results quickly, not only for individual careers, but also because there is an awareness that if the field does not deliver, the momentum to generate funding may be lost, restricting the further development of the field. There exists therefore a fine balance between the need to inflate promises of a new technology, and the need to moderate them, when the inflation of promises becomes damaging to reputations and to capital investments [17]. The dominant vision is part of this inflation of expectations, in order to build up excitement for the field.

Hypes around technological innovations can spur competition, coordinate activities and attract resources, but can also damage the credibility of scientific fields, professions, institutions and industry [17]. Besides harm to research participants and even potential patients, this can lead to a backlash for the field, with funding becoming more difficult to obtain, as was the case for gene therapy. Depending on different conditions of the hype-disappointment cycle, expectations can be refined or reoriented, making recovery after disappointment possible [15, 16].

The pressure to deliver results quickly, and the high competitiveness in the field of academic medicine can be at odds with responsible innovation [37]. To innovate responsibly, emerging technologies need to respond to societal needs and align with societal values, and stakeholders and the public need to be actively engaged from an early stage of the development process. Such a process needs time; moreover, many respondents believe there is still very little scientific knowledge available on epigenetics and the interaction with the 3D structure of DNA, transcription factors and the environment.

Despite agreement among scientists on the limiting scientific evidence of the dominant vision there are two ways that they navigate these uncertainties when reflecting on the current translation of epigenetic editing to the clinic. Some respondents emphasize that the current limitations in knowledge demand a slower pace of progression towards the clinic, preceded by more preclinical research and safety testing. Others articulate that knowledge of previous medical applications was also limited when going to the market. This includes gene editing, seen as less safe. As trial and error has always been part of the development of medicine, these respondents do not see why the bar would be set higher for epigenetic editing than for other emerging therapies. This analogy may be rhetorically effective but risks applying a double standard. Indeed, epigenetic editing is narrated as a targeted process: specific, predictable, more controllable, and less risky due to its potential reversibility. If this is the major advantage compared to existing therapies, then surely this advantage is (partially) dependent on the certainty of these traits. Dismissing the importance of the scientific evidence of these advantageous traits when going to the clinic, by referring to similar uncertainties of existing therapies at the time when they moved to the clinic, seems like a double standard.

### Future research: inclusivity and reflexivity

Analyzing the visions and expectations of researchers in emerging fields can uncover the values and assumptions that shape future technological innovation and uncover ideas of what constitutes their desirable future. Making these visions—and their underlying assumptions and actors—explicit allows reflection by a wider public, opening up possibilities for adjustment to foster responsible innovation. For the authors, our future research will be focused on citizen participation in discussions on epigenetic editing. The perceptions and visions of scientists, uncovered in this paper, will be a starting point for citizens to engage with this complex scientific issue, allowing the value of lay knowledge in decision-making [17]. Including more voices may allow for incorporating broader foresight and impact assessments for new technologies, extending beyond their expected market benefits and risks [22], with our aim of empowering citizens to reflect on the visions and assumptions of scientists (Verra *et al. in progress*). These initiatives are fruitful starting points for more inclusive decision-making.

Besides involving citizens, other stakeholders such as policy-makers [40] need to be involved in developing and shaping shared visions for epigenetic editing. In order to make well-informed decisions, it is important that these actors have a sufficiently complete picture of the innovation, the possible benefits, the uncertainties, the challenges and probably disadvantages of epigenetic editing.

Making the perspectives of scientists on epigenetic editing explicit, also allows for enhancing the reflexivity of scientists. In other research, we have conducted roundtables intended to (i) elicit an awareness among scientists of the limits and broader societal dimensions of their knowledge-practices; (ii) develop such an awareness while considering specific contexts of research, and (iii) anticipate the framing and evaluation of epigenetic developments in society [3]. These round table discussions were successful in providing a space for joint reflexivity for scientists.

### ELSI expectations

This study also sheds light on some of the ELSI that researchers consider important for the field of epigenetic editing technologies moving forward, including costs, accessibility and equity, and ethical limits to the use of epigenetic editing, such as human enhancement or human germline epigenetic editing.

Epigenetic editing-based therapies are expected to be costly, which raises concerns about limited or unequal access to these therapies by low-income individuals or in low-resource healthcare systems. High prices are often considered unfair when stakeholders – patients, experts, industry representatives – have divergent views on the value of novel therapies and on the level of uncertainty regarding their efficacy [38]. The use of epigenetic editing for the purposes of human enhancement, such as strengthening of athletic performance or reversing ageing, is not widely supported [39, 40]. Further, human germline epigenetic editing is believed to raise similar sets of ethical issues, notably, risks to future offspring, as does human germline gene editing [41]. However, researchers seem conflicted about the extent to which – unintended on- and off-target – epigenetic edits can be passed on to next generations and whether these pose risks for these generations, with many believing that they cannot, because of epigenetic reprogramming in the early embryo [23]. Either way, modifications that would affect the genome of human offspring, are prohibited in accordance with the Europe’s Convention on Human Rights and Biomedicine, commonly known as the Oviedo Convention^1^. These issues have not yet been widely discussed in the literature [42].

Although these are important matters to discuss, short term issues also need to be addressed to innovate responsibly. The roadmap from fundamental research to practical applications is strewn with moral decisions that require careful consideration. Further research should address ethical guidance for the design, conduct, and evaluation of clinical trials, including patient selection, risk-benefit assessment and informed consent.

### Plant science and medical science

We were surprised to find that the narrative about epigenetic editing differed between the plant scientists and the (fundamental) scientists in the medical field. Scientists in the medical field foresaw great potential for epigenetic editing, in both their own field, as well as the agricultural field, mainly because epigenetic editing does not change the DNA, with the assumption that the public would therefore look at it positively as opposed to how the public views genetically modified crops and their products. Most of them assumed that plant scientists would adopt the new technology. Plant scientists, however, did not believe that the public would view the subtle differences between epigenetic and genetic editing, and precisely because of their view of the perceived similarities between gene editing and epigenetic editing; for the latter, they assumed public acceptability would be low for epigenetically edited crops. Moreover, the techniques did not fulfill the requirements seed companies have. However, plant scientists did foresee a small role for the new technique in plant breeding, a larger role for research in plants, and a large role for epigenetic editing in the medical field.

## Conclusions

In this paper, we have studied the visions of scientists and industry that are co-shaping technologies, as well as their underlying values and assumptions. By making these visions explicit, they become a topic for scientists, policymakers, and the public to reflect on [20], and adapt or adjust, allowing for the co-creation of technological innovation. We have found that although experts are generally cautious about the benefits of future epigenetic applications, what is currently communicated to the outside world is restricted to the dominant narrative, without providing insights into the ways in which this narrative is questioned, and what visions for the future are contested among members of the field themselves. To allow for more inclusive decision-making and co-shaping the research field and future applications, it is important to improve reflexivity among researchers and to enable public engagement.

We urge the field to explicitly discuss what kinds of knowledge it deems necessary before moving epigenetic applications to the clinic or to be used for regulatory risk assessment. For this, a diverse group of scientists working towards epigenetic editing and those studying other aspects of the epigenome, among which basic researchers, should collaborate.

## Data Availability

The transcripts of the interviews used for the analysis in this paper are available from the corresponding authors on request.

## Footnotes

1
https://www.coe.int/en/web/human-rights-and-biomedicine/oviedo-convention (accessed 1 September 2025.
